# Prediction of Chronic Obstructive Pulmonary Disease Exacerbation Events by Using Patient Self-reported Data in a Digital Health App: Statistical Evaluation and Machine Learning Approach

**DOI:** 10.2196/26499

**Published:** 2022-03-21

**Authors:** Francis P Chmiel, Dan K Burns, John Brian Pickering, Alison Blythin, Thomas MA Wilkinson, Michael J Boniface

**Affiliations:** 1 School of Electronics and Computer Science University of Southampton Southampton United Kingdom; 2 my mHealth Limited Bournemouth United Kingdom; 3 National Institute for Health Research Applied Research Collaboration Wessex University of Southampton Southampton United Kingdom; 4 Faculty of Medicine University of Southampton Southampton United Kingdom

**Keywords:** COPD, machine learning, mHealth, exacerbation events, myCOPD, mobile health, digital applications, remote monitoring, chronic disease, digital health, health care applications

## Abstract

**Background:**

Self-reporting digital apps provide a way of remotely monitoring and managing patients with chronic conditions in the community. Leveraging the data collected by these apps in prognostic models could provide increased personalization of care and reduce the burden of care for people who live with chronic conditions. This study evaluated the predictive ability of prognostic models for the prediction of acute exacerbation events in people with chronic obstructive pulmonary disease by using data self-reported to a digital health app.

**Objective:**

The aim of this study was to evaluate if data self-reported to a digital health app can be used to predict acute exacerbation events in the near future.

**Methods:**

This is a retrospective study evaluating the use of symptom and chronic obstructive pulmonary disease assessment test data self-reported to a digital health app (myCOPD) in predicting acute exacerbation events. We include data from 2374 patients who made 68,139 self-reports. We evaluated the degree to which the different variables self-reported to the app are predictive of exacerbation events and developed both heuristic and machine learning models to predict whether the patient will report an exacerbation event within 3 days of self-reporting to the app. The model’s predictive ability was evaluated based on self-reports from an independent set of patients.

**Results:**

Users self-reported symptoms, and standard chronic obstructive pulmonary disease assessment tests displayed correlation with future exacerbation events. Both a baseline model (area under the receiver operating characteristic curve [AUROC] 0.655, 95% CI 0.689-0.676) and a machine learning model (AUROC 0.727, 95% CI 0.720-0.735) showed moderate ability in predicting exacerbation events, occurring within 3 days of a given self-report. Although the baseline model obtained a fixed sensitivity and specificity of 0.551 (95% CI 0.508-0.596) and 0.759 (95% CI 0.752-0.767) respectively, the sensitivity and specificity of the machine learning model can be tuned by dichotomizing the continuous predictions it provides with different thresholds.

**Conclusions:**

Data self-reported to health care apps designed to remotely monitor patients with chronic obstructive pulmonary disease can be used to predict acute exacerbation events with moderate performance. This could increase personalization of care by allowing preemptive action to be taken to mitigate the risk of future exacerbation events.

## Introduction

Chronic obstructive pulmonary disease (COPD) is a collection of progressive lung diseases, characterized by breathing difficulties and an irreversible reduction of lung function. It is one of the most prevalent chronic conditions in the world (in England, 2.19% of the population is expected to have a confirmed COPD diagnosis by 2030 [1]), and the absence of a cure means it represents a significant burden for patients who have to manage the condition on a daily basis [2,3]. A key characteristic of managing COPD is in mitigating the risk of “exacerbation events,” which can be defined as an acute sustained worsening of a patient’s condition that necessitates a change in medication or emergency care, including hospitalization [4]. Exacerbations accelerate lung function decline, and evidence suggests that the frequency of exacerbations increases with decreasing lung function [5-7]. Minimizing the number of exacerbation events can therefore have a significant impact on the prognosis for patients with COPD. Currently, several methods exist to help control exacerbation events, including pharmacological interventions, pulmonary rehabilitation, and self-management programs [8]. There is also an identified clinical need to predict exacerbation events in advance to personalize COPD treatment and offer the opportunity to provide targeted preemptive interventions [9,10].

In recent years, the advent of mobile health apps has facilitated increased remote management and care of patients with COPD [11,12]. These apps support the recording of temporally dense information about a patient’s condition, which allow (near) real-time monitoring of a patient’s symptoms, providing clinicians with a source of data to help them understand how the patient is managing their condition and gain an insight into the patient’s exacerbation frequency and severity. For the patient, digital health apps provide both an access point for educational content about their condition and the opportunity to improve their self-care, leading to better long-term management [13]. In the context of COPD, there is an opportunity to increase the efficacy of digital health apps further by leveraging the data they collect to predict acute exacerbation events and provide personalized alerts to the patient. These alerts could facilitate a clinically validated and personalized intervention program to mitigate the occurrence and reduce the severity of an acute exacerbation event.

In this report, we present a retrospective study making use of data collected by the myCOPD mobile app, a National Health Service–approved, clinically validated app for persons with a diagnosis of COPD [14]. This app assists with the management of COPD by providing educational content alongside a digital momentarily assessed symptom diary. Using this app, users self-report on the COPD-related symptoms they are currently experiencing as well as information that characterizes their long-term COPD status (ie, the COPD Assessment Test [CAT]). Using statistical analysis and machine learning methods, we evaluate the effectiveness of exploiting this simple self-reported information to predict exacerbation events in the near future and discuss how such predictions could be used to improve the long-term outcomes for people with COPD.

## Methods

### Data Set Description

**Figure 1:**
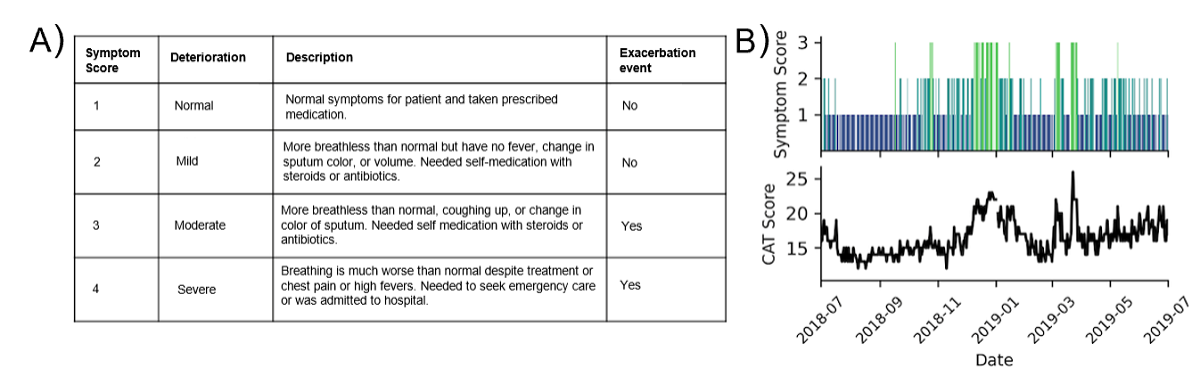
Self-reported symptom scores and chronic obstructive pulmonary disease assessment test (CAT) scores. (A) Symptom score rankings and classification of whether this score corresponds to an exacerbation event, as defined in the context of this work. (B) Example user (with high reporting frequency) self-reporting timeline where the top panel displays self-reported symptom scores and the bottom panel self-reported CAT results. CAT: chronic obstructive pulmonary disease assessment test.

We used an anonymized extract of daily user self-reports submitted to the myCOPD app between January 1, 2017 and December 31, 2019 (inclusive). All users of the myCOPD app are clinically diagnosed with COPD, with app usage limited to patients “prescribed” the app by clinicians as part of agreed care plans. A single report features a self-assessed symptom score (Figure 1), which is a 4-point scale ranging from normal symptoms to a severe deterioration of symptoms requiring medical intervention, including hospitalization. We encode symptoms as an ordinal variable between 1 and 4, indicating increasing severity of COPD-related symptoms (Figure 1A). Symptom scores have a level of subjectivity across users (eg, the severity of symptoms considered normal for a given user will vary) with users provided with education to increase awareness and understanding of what baseline symptom scores would be considered normal for them. Users also perform a CAT at regular (approximately monthly) intervals. The CAT is an 8-question assessment and yields a score between 0 and 40, where higher values indicate a more severe impact of COPD on a user’s overall health [15]. CAT is a validated and an accepted way of quantifying the burden of COPD on someone’s life [16,17]. The reporting frequency of symptoms and CAT scores for our cohort is presented in Table S1 in Multimedia Appendix 1. In addition to these scores, our data set also features additional demographic and lifestyle information self-reported to the app. These include patient age, gender, current smoking status, and the number of years they have been smoking for.

### Ethics Approval and Data Governance

This work received ethics approval from the University of Southampton’s Faculty of Engineering and Physical Science Research Ethics Committee (ERGO/FEPS/52137) and was reviewed by the University of Southampton Data Protection Impact Assessment panel (DPIA 0045), with the decision to support the research.

### Defining Exacerbation Events

We use a symptom-based definition for exacerbation events where an event is marked to have occurred if a patient self-reports a score of 3 or 4 corresponding to a moderate or severe deterioration, respectively, from a patient’s normal symptoms. A score of 3 indicates that a patient is more breathless than normal, coughing up sputum or with change in sputum color, and has needed to self-medicate using steroids or antibiotics. A score of 4 indicates that a patient’s breathing is much worse than normal despite treatment, has chest pain or a high fever, and has needed to seek emergency care or was admitted to the hospital (Figure 1) [13].

### Cohort Selection and Data Set Segregation

**Figure 2:**
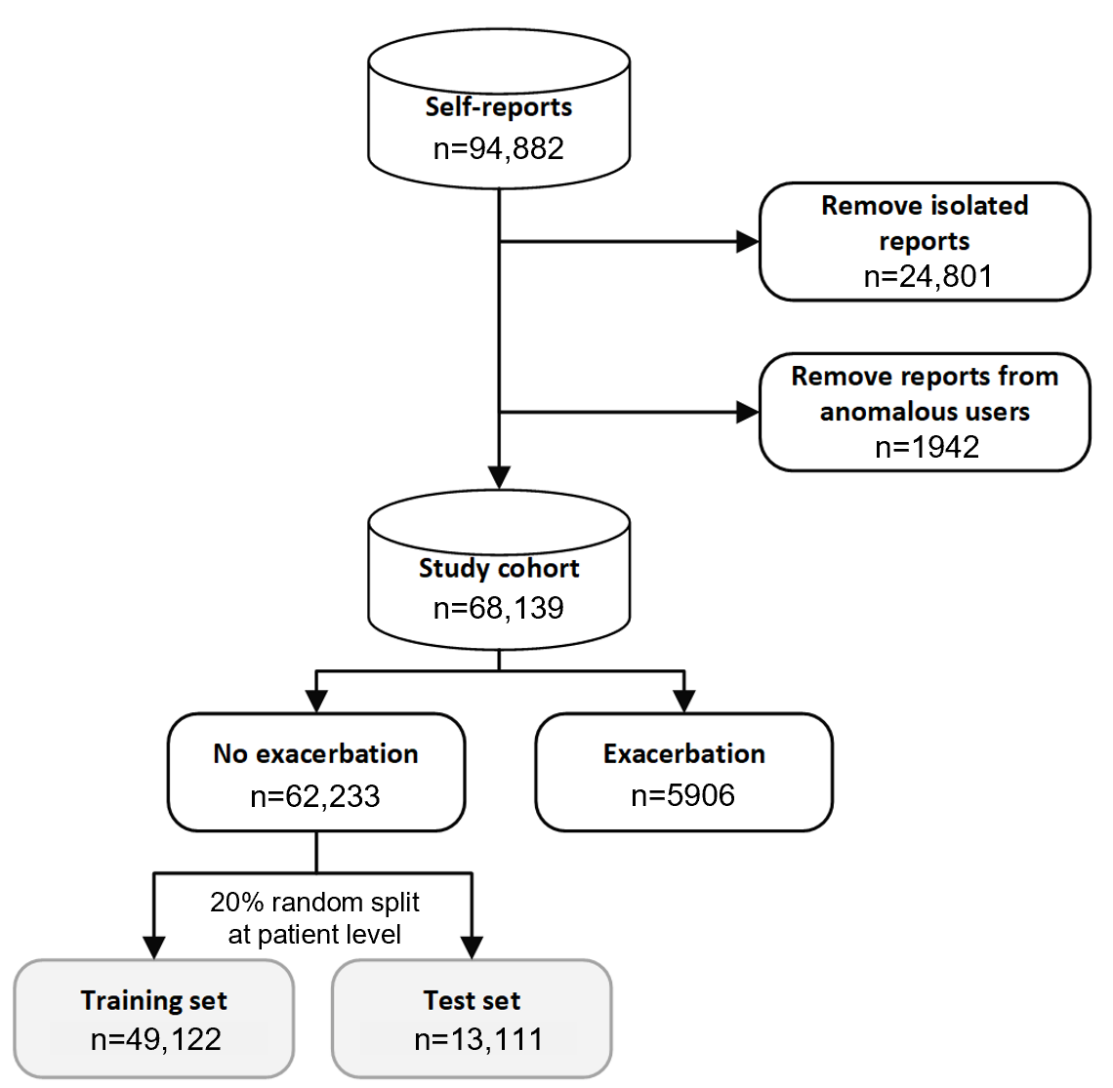
Selection of self-reports in our study cohort containing 2374 patients. Isolated reports (n=24,801) were those without a subsequent report in the following 3 days. Anomalous users (n=1942) were those who only reported exacerbation events or self-reported to the myCOPD app before their registration date. Exacerbation events (n=5906) were all self-reported to the app by 742 patients.

In total, 5170 users were included in the extract who reported a total number of 94,882 reports in the study period. User registration was incremental (ie, not all users registered at the same time) throughout the period of the study, and self-reports were not necessarily submitted every day. To create our study cohort, we followed a selection process outlined in Figure 2. First, isolated symptom reports (those in which a second report was not made within 3 days) were removed because the target variable could not be reliably calculated. Next, reports from anomalous users (those only reporting exacerbation events or entering self-reports before their registration date) were removed. After removal of these reports, we obtained our final study cohort featuring 68,139 self-reported symptom scores from 2374 unique users. Patient information relates to the time at which the patient first reported to the app (eg, if there smoking status changed, Table 1 summarizes the first reported status). Table 1 presents the characteristics of 2374 unique patients in our cohort (including patients in both train and test). For our user cohort, the mean reporting frequency between patients’ first and last reports to the app was 3.28 symptom score reports per week and 0.68 CAT score reports per week.

From our cohort of 2374 users, 1672 users were between the ages of 60 years and 79 years inclusive (Table 1). Only 650 users reported their gender, with 419 males reporting compared to 231 females. A large fraction (n=1157) of users reported their smoking history, with 86.5% (1001/1157) of those reporting being either a current or ex-smoker (Table 1). Out of the self-reports included in this study, 742 patients reported 5906 self-reports that correspond to an exacerbation event, corresponding to 8.7% (5906/68,139) of the total reports and 31.3% (742/2374) of the patients (Figure 2). The median number of exacerbation event reports per patient was 3 (IQR 1-7) for our cohort.

**Table 1:** Patient demographics and smoking status in our cohort (N=2374). All information was self-reported to the myCOPD app.

Group, subgroups			Patients, n (%)
**Age group (years)**			
	Missing	10 (0.4)	
	19-29	7 (0.3)	
	30-39	39 (1.6)	
	40-49	89 (3.7)	
	50-59	325 (13.7)	
	60-69	791 (33.3)	
	70-79	881 (37.1)	
	80-89	212 (8.9)	
	90-99	15 (0.6)	
	100-110	5 (0.2)	
**Gender**			
	Missing	1724 (72.6)	
	Male	419 (17.6)	
	Female	231 (9.7)	
**Smoking status**			
	Missing	1217 (51.3)	
	Ex-smoker	843 (35.3)	
	Nonsmoker	156 (6.6)	
	Smoker	158 (6.7)	

### Predicting Exacerbation Events

For each daily self-report, we created a binary variable that indicated whether a report is followed by an exacerbation event in the following 3 days. A 3-day window was chosen empirically based on clinical guidance to be close enough to the future exacerbation event for any signal to be present in the data but sufficiently far from the event such that a range of preemptive actions could be available to patients. For the training of the prognostic models, we selected only reports in which the patient did not report an exacerbation event on the same day (n=49,122, Figure 2). We then randomly assigned reports from 19.2% (13,111/68,139) of the patients to a holdout test set (Figure 2).

We created a baseline heuristic model that uses only a user’s most recently reported symptom score. The model assigns users to 2 risk groups: users reporting a symptom score of 1 are predicted to be at low risk of exacerbation (1.7% risk) within 3 days, and users reporting a symptom score of 2 are predicted to be at heightened risk (7.2% risk) of exacerbation within 3 days. Percentages in brackets correspond to the mean 3-day exacerbation rate for all reports in the training set with symptom scores of 1 or 2, respectively. The heuristic model is equivalent to a decision tree with a depth of 1. Supervised machine learning models make use of patient demographics, lifestyle information, self-reported information, and aggregate features that summarize a patient’s (recent) self-reporting history. A full schema of variables used by our models is presented in Table S2 of Multimedia Appendix 1. We used logistic regression with regularization and a random forest classifier each trained by 5-fold and grouped cross-validation at the user level, that is, reports from a single user appear exclusively in either the training or validation fold. Missing CAT scores were forward-filled imputed at the user level where possible. All other missing values were filled using mean imputation within fold. Either target or ordinal encoding was used for all categorical variables (Table S2 in Multimedia Appendix 1). Model hyperparameters were optimized on the out-of-fold validation samples by Bayesian optimization via the Tree Parzen Estimator algorithm as implemented in the HyperOpt Python library [18,19]. Model performance was evaluated on the holdout test set, and 95% CIs were estimated by bootstrapping. To create a binary decision of exacerbation risk, model predictions were dichotomized with thresholds chosen to yield either a fixed specificity or the maximum Youden’s J statistic on the test set [20].

## Results

### Relationship Between Self-reported Scores and Exacerbation Events

**Figure 3:**
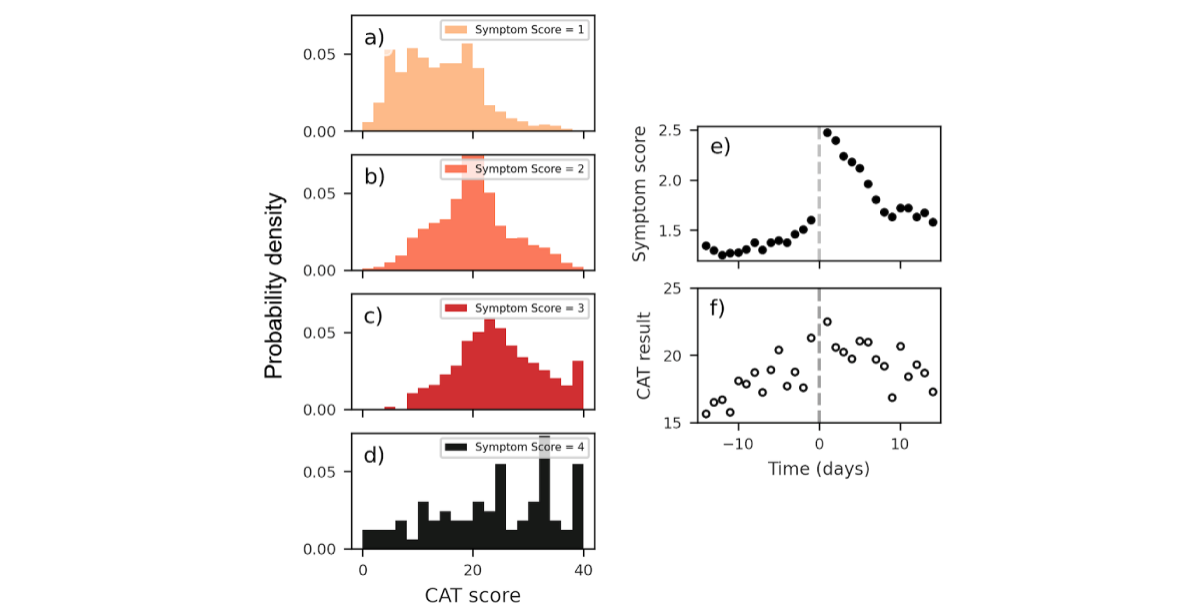
Self-reported symptom scores and results of chronic obstructive pulmonary disease assessment test (CAT) for reports in our 2374 patient cohort. (A-D) Displays the self-reported CAT result stratified by the self-reported symptom score (row) on the day of test completion. (E) Mean self-reported symptom scores in the days preceding (and following) a day where a patient self-reports their first exacerbation event. (F) Mean self-reported result of CAT in the days preceding (and following) a day where a patient self-reports their first exacerbation event. Grey dashed lines in all panels highlight the day of the first reported exacerbation event (time=0 days). Panels E and F indicate that exacerbation events can be associated with a worsening of symptom scores and CAT results several days in advance of the event. The width of the observed peaks (see panel E, right of dashed line) following the start of the exacerbation event demonstrates that exacerbation events can be multiple day events. CAT: chronic obstructive pulmonary disease assessment test.

Figure 3 investigates the relationship between symptom scores and CAT scores self-reported to the myCOPD app and self-reported exacerbation events. Panels A to D of Figure 3 display the correspondence between symptom scores and the CAT results when self-reported on the same day. Although for each symptom score, users nearly report the full range of CAT scores, there is a clear correlation between CAT scores and symptom scores, with users reporting higher symptom scores more likely to also report a higher CAT score. For example, the mean CAT score reported when a user reports a symptom score of 1 is 13.5, which is significantly lower (*P*<.001) than the mean CAT score (19.5) when a user reports a symptom score of 2. Such a correlation is to be expected; research has shown increased CAT scores correlate with an increased exacerbation frequency [21], which in turn would lead to higher symptom scores being reported in our study.

In Figures 3E and F, we evaluate the sensitivity of symptom scores and CAT results to future exacerbation events. We display how the mean of the 2 reported variables change in days preceding (and following) the day in which users self-report their first exacerbation event to the app (not necessarily their first ever exacerbation event). By inspection of Figure 3E, we see that the mean reported symptom score in proximity of the first reported exacerbation event increases, indicating that in the days preceding their first exacerbation event, users are increasingly likely to report a mild deterioration of symptoms (symptom score of 2). Changes in the mean symptom score calculated across all users can be seen several days in advance, suggesting that at least some users are observing a mild deterioration in symptoms several days in advance of exacerbation events. For days subsequent to users’ first self-reported exacerbation (right of the dashed line in Figure 3E), the mean symptom score initially exceeds 2, showing that self-reported exacerbation events can be multiday events—consistent with the current understanding of exacerbation events [4]. Similarly, in Figure 3F, the reported mean CAT result is observed to increase in magnitude in the days preceding an exacerbation event and then decrease (at a slower rate) following a reported event. Overall, these trends indicate there is potential in using these self-reported variables to predict at least a subset of exacerbation events in advance.

### Predicting Exacerbation Events

**Figure 4:**
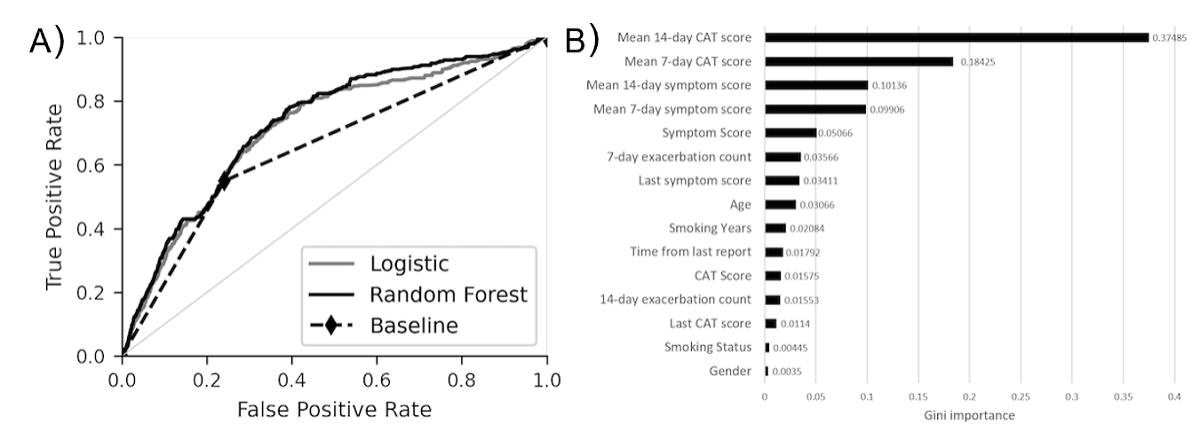
Model performance evaluated on the patient holdout test set. (A) Receiver operating characteristic curve of our models. The baseline model (dashed line) has only 1 nontrivial threshold for dichotomizing the prediction (diamond marker), whereas the machine learnt models has a number of possible thresholds, which needs to be optimized to suit the use case (so called sensitivity-specificity trade-off). (B) Feature importance (Gini importance) for the random forest model. CAT: chronic obstructive pulmonary disease assessment test.

Figure 4 shows the receiver operating characteristic (ROC) curve comparing a set of prognostic models to predict exacerbation. This includes a baseline model alongside the 2 machine learning models—a logistic regression model (solid grey line) that does not consider variable interactions and a random forest classifier (solid black line) that does. The baseline prognostic model captures the key feature about exacerbation events observed in our data: persons reporting a mild deterioration of symptoms are significantly (*P*<.001) more likely to experience an exacerbation event in the next 3 days compared to those reporting normal symptoms, with a relative risk of 4.16 (95% CI 3.8-4.5). On the holdout test set, the baseline model obtained an area under the receiver operating characteristic (AUROC) of 0.655 (95% CI 0.676-0.689). The logistic regression model obtained an AUROC of 0.697 (95% CI 0.689-0.711) and the random forest model 0.727 (95% CI 0.720-0.735) on the holdout test (Table 2). The significantly higher (*P*<.001) performance of the random forest model suggests either interactions between variables are important in discriminating between reports associated with exacerbation within 3 days or nonlinear relations are present.

In Figure 4B, we present the feature importance (Gini importance) for our random forest model. The most important features are the patients’ recent CAT scores (mean 14-day CAT score and mean 7-day CAT score), consistent with research that showed CAT scores are an effective way of quantifying the severity of a patient’s COPD, which in turn, is linked to their exacerbation risk [16,17]. The next most important features are those quantifying patients’ recently reported symptom scores. Symptom scores reflect the symptoms a patient is (or was recently) experiencing, and we have shown (Figure 3E) that people reporting higher symptom scores are more likely to report an exacerbation event within 3 days compared to those reporting lower symptom scores. It, therefore, is reasonable that the machine learning model can use this information to better quantify a patient’s exacerbation risk.

In Table 2, we present the sensitivity and specificity of the baseline model and the machine learning models evaluated on the holdout test set. Although the baseline model is already dichotomized, for the machine learning models, a threshold must be chosen to binarize the continuous exacerbation risks they produce. The baseline model obtained a sensitivity of 0.551 (95% CI 0.508-0.596) with specificity of 0.759 (95% CI 0.752-0.767). Although neither machine learning model significantly outperforms the baseline model at the same specificity (eg, compare models A and E in Table 2), the tuning of the threshold used to dichotomize the machine learning model predictions can lead to a range of sensitivities and specificities (compare models C, D, and E in Table 2) on the holdout test, which could be tuned to match different escalation policies and interventional strategies. For example, the random forest model can be tuned to yield a sensitivity of 0.921 (95% CI 0.907-0.935) or 0.576 (95% CI 0.553-0.594) with respective specificities of 0.250 (95% CI 0.246-0.254) or 0.750 (95% CI 0.749-0.751).

**Table 2:** Model performances evaluated on the holdout test set.^a^

Name	Model	Area under the receiver operating characteristic curve (95% CI)	Threshold	Sensitivity (95% CI)	Specificity (95% CI)
A	Baseline model	0.655 (0.632-0.676)	N/A^b^	0.551 (0.508-0.596)	0.759 (0.752-0.767)
B	Logistic regression	0.697 (0.689-0.711)	Youden’s J statistic	0.708 (0.625-0.768)	0.644 (0.574-0.706)
C	Random forest	0.727 (0.720-0.735)	Youden’s J statistic	0.755 (0.676-0.813)	0.629 (0.564-0.700)
D	Random forest	0.727 (0.720-0.735)	Specificity=0.25	0.921 (0.907-0.935)	0.250 (0.246-0.254)
E	Random forest	0.727 (0.720-0.735)	Specificity=0.75	0.576 (0.553-0.594)	0.750 (0.749-0.751)

^a^The area under the receiver operating characteristic curve column denotes the area under the receiving operator curve (Figure 4) for each model. The 3 rightmost columns display the sensitivity and specificity of models at predicting exacerbations with different thresholds used to dichotomize the predictions. The baseline model is already binary and only has 1 nontrivial configuration, but the threshold used to dichotomize the machine learning models (B-E) can be tuned to suit the intended context of the model. The maximum of Youden’s J statistic is used as a baseline criterion for dichotomizing the prediction (models B and C), and other cutoffs yielding fixed specificities are investigated for the random forest model. The area under the receiver operating characteristic curve for models C, D, and E are the same since they correspond to the same underlying model.

^b^N/A: not applicable.

## Discussion

The etiology of COPD exacerbations is now well understood, with bacterial and viral infections, exposure to extreme weather, and air pollution being the key drivers set against the background of poor disease control. Effective treatments are available for COPD, with early recognition of symptoms of deterioration and prompt intervention having been shown to be associated with better outcomes [22]. Current models of care for people living with COPD include the use of regular inhaled medications and rescue packs of antibiotics and steroids that they keep at home on standby for use during exacerbations. Patients are expected to initiate treatments if they suspect they have an exacerbation. To influence clinical outcome beneficially, a predictive model needs to enable a preemptive intervention with the likely impact maximized the earlier this occurs. In the context of COPD, exacerbation interventions may include increased use of inhaled medication or additional administration of rescue packs of oral antibiotics and corticosteroids [23]. The current standard of care has been for patients to take these treatments when the symptoms are exacerbating, which creates a reactive model of care requiring significant clinical deterioration to have occurred before an intervention is started. Our own work has shown that early treatment of exacerbations is associated with improved clinical outcomes, including faster recovery times [24]. As such, there is, however, a strong evidence base to suggest that this paradigm of care is inadequate and leads to increasing numbers of hospital admissions, unscheduled visits to primary care, and prolonged episodes of ill health and sickness absence from work.

Early prediction of COPD exacerbation events has the potential to change clinical practice and transform management of COPD. With the preemptive warning of a future exacerbation, the new app codeveloped with patients with COPD will alert patients of the risk and provide information on appropriate therapy options. More effective treatment of exacerbations offers the possibility of faster recovery, less relapses, and overall, therefore, better health-related quality of life, improved disease control, and potentially fewer exacerbations. Our study has shown that symptom information self-reported to the myCOPD app displayed correlation to the start of the future exacerbation events (Figure 2E), and we found that machine learning models utilizing this information and other sources of self-reported data were able to identify patients at risk of exacerbation within 3 days with moderate discriminative ability (AUROC 0.727, 95% CI 0.720-0.735). Therefore, if presented appropriately, this risk prediction model could enable patients to self-manage more effectively by intervening before life-threatening inflammation and infection can become established.

Various approaches to continuous remote monitoring of patients with COPD in communities have emerged in recent years that use sensor technologies to measure physiological parameters (respiratory rate, pulse oximetry, spirometry, blood pressure, weight, etc) and physical activity [25]. The consequence is that many of the symptoms and parameters of COPD exacerbation [26] previously measured in clinical settings can now be measured at home. This trend is transforming decision support from tools used by clinicians to tools empowering patients in everyday life. Traditionally, such tools are developed using predefined rules applied to population-based thresholds on parameters, as per our baseline model. However, new approaches are needed to support a care paradigm shifting to remote monitoring of complex parameters with varying degrees of reporting compliance, data quality and patient condition, and behaviors.

Machine learning models are well positioned to address these requirements because they can dynamically learn complex nonlinear relations between variables, which are inaccessible to handcrafted models, and despite the complexity of the models, they can be easily integrated into digital health apps such as myCOPD. This greatly facilitates the potential uptake of the model since they can be directly implemented in an active digital ecosystem for patient care that fosters data acquisition and can position predictions within new models of patient-to-clinician interaction with predictions updated every time a user provides new data. For machine learning models, it is important to match their configuration to the escalation policy. If models are used as a binary alert system, this is achieved by analyzing the (so-called) sensitivity-specificity trade-off, considered in Table 2 or Figure 4. In Table 2, 3 configurations of the random forest model are chosen (models C, D, and E), which yield different sensitivities and specificities. For example, model D in Table 2 uses a threshold chosen to obtain a specificity of 0.25 on the test set and achieves a sensitivity of 0.921 (95% CI 0.907-0.935). This configuration could be appropriate if false positives are not of significant concern (eg, if the prescribed intervention plan is of little risk to the patient). Ultimately, different configurations allow flexibility in the resulting escalation policies and is the key advantage of the machine learning models compared to the baseline model. Our use of decision tree–based algorithms offers high explainability necessary for interpretation and transparency in predictions. Single decision tree classifiers are directly explainable and provide decision support in a manner similar to classical clinical decision support tools, while ensemble methods can be made explainable by taking advantage of recent advancements (eg, Shapley additive explanations [27]).

The predictive performance of the machine learning models for COPD exacerbation can be improved by adding COPD-related variables. Health and lifestyle activity factors (including comorbidity and socioeconomic status) are believed to impact COPD exacerbation frequency [22]. These could be acquired from a patient’s medical record or collected with questionnaires and used by the algorithm to further refine its predictions. Additionally, since self-reported deterioration in symptoms can occur several days before an exacerbation (Figure 2E), it is reasonable that symptom information could be collected in a more granular manner in the days preceding an exacerbation event. This could be achieved with medical devices (eg, smart inhalers) that automatically incorporate spirometry, wearables designed to monitor a person’s lung function, and COPD-related physiological and behavioral variables (eg, oxygen saturation, respiration rate, temperature) or through more granular self-reporting of symptoms through the digital app [28].

Reporting compliance is a concern for safety, effectiveness, and acceptance of models. There is a need to ensure the burden of technology is reduced to a minimum, that incentives and benefits of reporting are aligned, and where appropriate, automatic observations and measurements are used to capture data (eg, smart inhalers). Our self-reports were collected in a prospective fashion by using momentary assessments of a patient’s COPD symptoms. Reporting compliance and individual variation in reporting behaviors could still be detrimental to our results, with exacerbation frequencies or severity being misreported [29]. Our decision to remove isolated reports outside of the 3-day window may introduce bias owing to variations in patient reporting behaviors and the possibility of underrepresentation of exacerbating symptoms in patients who may be too ill to report at sufficient frequency, especially those admitted to the hospital. Integration with medical records would address this concern by providing the ground truth for severe exacerbation where patients require urgent medical care. Imperfect reporting compliance also acts to limit the amount of data available to our machine learning model, which may limit their predictive ability. Although accountability to clinicians may improve compliance for some patients, ideally what is required is trust, acceptance, and engagement by those people living with COPD. Therefore, our model must be integrated into the digital health platform with patient groups participating in the co-design and optimization of the intervention to identify barriers to intervention and target behaviors prior to and during a clinical trial [30].

To conclude, our results suggest that data self-reported to a digital health app, designed for the management of people with COPD, can be used to identify users at risk of exacerbation within 3 days with moderate discriminative ability (AUROC 0.727, 95% CI 0.720-0.735). Further research utilizing additional linked data (particularly from medical devices such as smart inhalers, physiological monitoring sensors, and environmental sensors) are expected to increase the accuracy of these models.

## Appendix

Multimedia Appendix 1 Supplementary data.

## Abbreviations

AUROC: area under the receiver operating characteristic curve
CAT: chronic obstructive pulmonary disease assessment test
COPD: chronic obstructive pulmonary disease

## Data Availability

Data will be made available upon reasonable request to persons with a university affiliation. Requestors will need appropriate data protection, governance, and ethical review in place.
