# Supplementary material for: Prediction of Chronic Obstructive Pulmonary Disease Exacerbation Events by Using Patient Self-reported Data in a Digital Health App: Statistical Evaluation and Machine Learning Approach
Source: JMIR Med Inform. 2022 Mar 21;10(3):e26499. doi: 10.2196/26499 (PMC8981014; doi:10.2196/26499)
Supplement: Multimedia Appendix 1 [file medinform_v10i3e26499_app1.docx]

**Multimedia Appendix 1**

**Table S1.**Number of reports of given symptom and CAT scores for our study cohort. Users are not requested to perform a CAT every day but approximately at monthly intervals, although users can perform them more regularly if desired. In our prognostic models missing CAT scores are forward-filled imputed across reports from the same user.

|  | | Number of reports, n (%) |
| --- | --- | --- |
| **Symptom score** | | |
|  | 1 | 46,695 (68.5) |
|  | 2 | 15,538 (22.8) |
|  | 3 | 5534 (8.1) |
|  | 4 | 372 (0.5) |
| **CAT^a^ score** | | |
|  | 0-4 | 1121 (1.6) |
|  | 5-9 | 1984 (2.9) |
|  | 10-14 | 2142 (3.1) |
|  | 15-19 | 3031 (4.4) |
|  | 20-24 | 1700 (2.5) |
|  | 25-29 | 761 (1.1) |
|  | 30-34 | 492 (0.7) |
|  | 35-40 | 191 (0.3) |

^a^CAT: Chronic obstructive pulmonary disease Assessment Test.

**Table S2.** Schema of all variables used by our prognostic models.

| Variable | Data Type | Encoding | Description |
| --- | --- | --- | --- |
| Age | Integer | N/A^a^ | User age at time of registration |
| Gender | String | Ordinal | User gender |
| Symptom score | Integer | Ordinal | Reported symptom score (1,2,3,or 4) |
| CAT^b^ score | Integer | N/A | Reported CAT score (1,2,3,or 4) |
| Smoking status | String | Target | One of: Smoker, Ex-smoker, Non-smoker |
| Smoking years | Integer | N/A | Number of years smoked |
| Time from last report | Integer | N/A | Time (in days) since the user last reported |
| Last symptom score | Integer | N/A | User’s last reported symptom score |
| Last CAT score | Integer | N/A | User’s last reported CAT score |
| Mean 7-day symptom score | Float | N/A | Mean symptom score for user over last 7 days |
| Mean 7-day CAT score | Float | N/A | Mean CAT score for user over last 7 days |
| 7-day exacerbation count | Integer | N/A | Number of days on which user reported an exacerbation event over the last 7 days |
| Mean 14-day symptom score | Float | N/A | Mean symptom score for user over last 14 days |
| Mean 14-day CAT score | Float | N/A | Mean CAT score for user over last 14 days |
| 14-day exacerbation count | Integer | N/A | Number of days on which user reported an exacerbation event over the last 14 days |

^a^N/A: not applicable.

^b^CAT: Chronic obstructive pulmonary disease Assessment Test.
